# CRISPR-Cas systems as next-generation antimicrobials: a systemic review of mechanisms, delivery strategies, and translational challenges

**DOI:** 10.3389/fmicb.2026.1747931

**Published:** 2026-05-11

**Authors:** Maha S. I. Wizrah

**Affiliations:** Department of Biology, College of Science and Humanities in Al-Kharj, Prince Sattam Bin Abdulaziz University, Al-Kharj, Saudi Arabia

**Keywords:** anti-CRISPR, antimicrobial resistance, CRISPR–Cas, multidrug resistance, phage delivery, plasmid curing, synthetic biology

## Abstract

**Introduction:**

The rapid global increase in multidrug-resistant (MDR) bacteria has compromised the effectiveness of conventional antibiotics, stressing the urgent need for alternative antimicrobial strategies. CRISPR–Cas systems, originally evolved as bacterial adaptive immune mechanisms, provide programmable and highly specific tools for targeting antimicrobial resistance (AMR) determinants.

**Objective:**

This systematic review aims to evaluate the antibacterial mechanisms, delivery strategies, preclinical evidence, safety considerations, and translational potential of CRISPR–Cas systems for combating MDR bacterial infections.

**Methods:**

A systematic literature search was conducted in PubMed, Scopus, Cochrane Library, and Web of Science up to January 2026 in accordance with PRISMA 2020 guidelines. Eligible studies included original *in vitro* and *in vivo* experimental or preclinical investigations assessing CRISPR–Cas systems (Cas9, Cas12, Cas13, or related effectors) for antibacterial activity or antibiotic resensitization. Data were extracted on CRISPR effector type, bacterial target, delivery platform, and therapeutic outcome. Due to methodological heterogeneity, results were synthesized narratively.

**Results:**

Most studies reported effective killing or resensitization of MDR bacteria through chromosomal double-strand break induction, resistance plasmid curing, integron disruption, or RNA-targeted cleavage. Cas9 was the most frequently employed effector, followed by Cas12 and Cas13. Delivery strategies included bacteriophages, conjugative plasmids, and nanoparticle-based systems, with phage-mediated delivery demonstrating the most consistent efficacy in complex environments and animal models. Notably, a CRISPR-enhanced engineered bacteriophage cocktail (LBP-EC01) has advanced to clinical evaluation.

**Discussion:**

Overall, the evidence supports CRISPR–Cas antimicrobials as a promising precision-based approach for addressing AMR. However, major barriers remain, including limited host range, instability in physiological environments, emergence of escape mutations, and insufficient data on off-target effects and long-term safety. Addressing these challenges through optimized delivery platforms, multiplex targeting strategies, and standardized safety and regulatory frameworks will be essential for clinical translation.

**Systematic review registration:**

https://www.crd.york.ac.uk/PROSPERO/view/CRD420261319789, identifier CRD4201319789.

## 1 Introduction

Antimicrobial resistance (AMR) has been recognized by the WHO as one of the biggest global health threats. The rising incidence of drug-resistant (MDR) and extensively drug-resistant (XDR) bacterial pathogens (e.g., *Enterobacterales*, *Pseudomonas aeruginosa*, *Acinetobacter baumannii*, *Staphylococcus aureus*) has emerged sooner than the development of new antibiotics (Mancuso et al., 2021). Conventional treatments are progressively losing effectiveness, especially against hospital- and community-acquired infections (CAIs), which occur outside healthcare settings and are commonly caused by viruses such as influenza and COVID-19 or bacteria like *Streptococcus pyogenes*, as well as biofilm-associated infections and infections in immunocompromised individuals (Marino et al., 2025).

CRISPR–Cas (Clustered Regularly Interspaced Short Palindromic Repeats and CRISPR-associated proteins) systems are bacterial and archaeal adaptive immune systems that defend against mobile genetic elements (MGEs) such as phages and plasmids (Barrangou and Marraffini, 2014; Koonin and Makarova, 2017). Their programmability and specificity make them promising agents for antimicrobial use, capable of killing specific pathogens, eradicating resistance genes, or inhibiting virulence with minimal impact on commensal flora (De La Fuente et al., 2024; Mayorga-Ramos et al., 2023). Recent advances in CRISPR biology, delivery systems, and anti-CRISPR regulation have opened new opportunities for precise genome targeting against multidrug resistant bacteria.

This research question is: How effective and translationally feasible are CRISPR–Cas systems (Cas9, Cas12, Cas13, and related effectors) as antibacterial tools for combating multidrug-resistant (MDR) bacteria, in terms of their mechanisms of action, delivery platforms, preclinical outcomes, and associated limitations?

## 2 Methods

This systematic review was conducted according to the PRISMA 2020 guidelines for transparent reporting of systematic reviews.

### 2.1 Eligibility criteria

The review followed the PICOS (Population, Intervention, Comparator, Outcomes, and Study Design) framework to maintain consistency and clarity.

Population (P): Bacterial species exhibiting antimicrobial resistance (AMR), multidrug resistance (MDR), or extensive drug resistance (XDR), including *Enterobacterales, Pseudomonas aeruginosa, Acinetobacter baumannii, Staphylococcus aureus*, and other clinically relevant pathogens.

Intervention (I): Application of CRISPR–Cas systems such as Cas9, Cas12, Cas13, and related effectors for antibacterial activity, elimination of resistance genes, biofilm disruption, or virulence attenuation.

Comparator (C): Conventional antimicrobial agents, bacteriophage therapy, or untreated controls used to assess relative efficacy and specificity.

Outcomes (O): Reduction in bacterial viability or resistance gene carriage, restoration of antibiotic susceptibility, inhibition of biofilm formation, attenuation of virulence, and safety or off-target evaluations.

Study Design (S): Original *in vitro* and *in vivo* experimental studies, including preclinical animal models, assessing antibacterial mechanisms or therapeutic effects of CRISPR–Cas systems.

### 2.2 Information sources and search strategy

A comprehensive literature search was conducted in PubMed, Scopus, Cochrane Library, and Web of Science databases up to January 2026. The search strategy combined controlled vocabulary and free-text terms such as “CRISPR-Cas systems,” “antimicrobial resistance,” “bacteriophage,” “nanoparticles,” and “drug-resistant bacteria,” using Boolean operators (AND/OR). Reference lists of selected articles and reviews were manually screened to identify additional relevant studies.

### 2.3 Inclusion and exclusion criteria

Studies were included if they:

1. Investigated CRISPR–Cas systems for antibacterial or antimicrobial purposes (e.g., chromosomal targeting, plasmid curing, biofilm inhibition).
2. Focused on bacterial pathogens associated with AMR or MDR.
3. Reported experimental data from *in vitro*, *in vivo*, or preclinical models.
4. Were peer-reviewed and published in English up to 2026.

Studies were excluded if they focused on non-bacterial applications, lacked antimicrobial relevance, or were not peer-reviewed (such as conference abstracts, commentaries, or editorials).

### 2.4 Study selection

**FIGURE 1:**
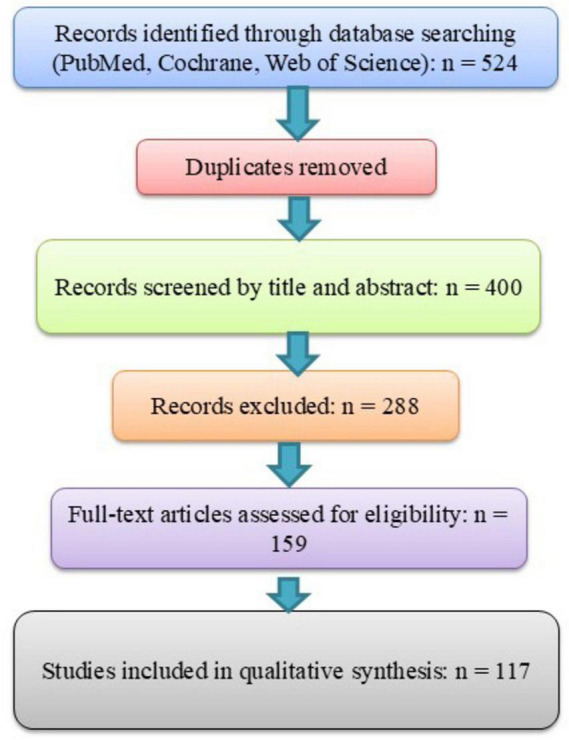
PRISM 2020 flow diagram for study selection. This figure outlines the systematic process used for literature identification, screening, eligibility assessment, and inclusion in the review. A total of 524 records were retrieved through database searches (PubMed, Cochrane, and Web of Science). After removing duplicates, 400 records were screened by title and abstract, with 288 excluded for not meeting the inclusion criteria. The remaining 159 full-text articles were assessed for eligibility, and 117 studies were finally included in the qualitative synthesis.

All identified records were imported into a reference management tool; here we used Zotero, for duplicate removal. A total of 524 records were identified across databases. After removal of duplicates, 400 studies were screened by title and abstract, with 288 excluded for irrelevance. The remaining 159 full-text articles were assessed for eligibility, and 117 studies were included in the final qualitative synthesis (Figure 1).

**FIGURE 2:**
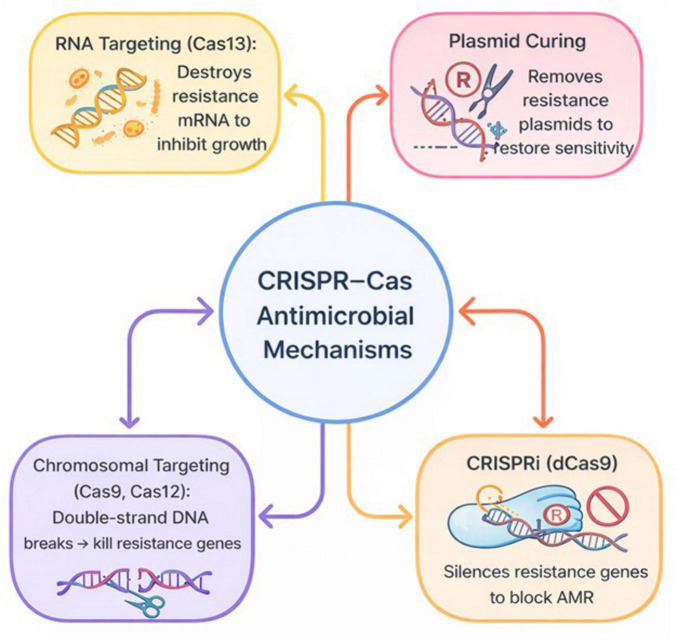
Mechanism of CRISPER-Cas antimicrobial action. The main mechanisms by which CRISPR–Cas systems combat multidrug-resistant (MDR) bacteria. These include chromosomal targeting using Cas9 or Cas12 to induce double-strand DNA breaks in essential or resistance genes, leading to bacterial death or resensitization; plasmid curing, which removes antibiotic resistance plasmids such as *bla*, *mcr-1*, and *vanA*, thereby restoring antibiotic susceptibility; integron targeting (e.g., *intI1*) to collapse multidrug resistance cassettes; transcriptional repression via CRISPR interference (dCas9) to silence resistance or virulence genes without DNA cleavage; and RNA targeting using Cas13 to degrade resistance or virulence transcripts, inhibiting bacterial growth. Together, these complementary strategies demonstrate the versatility and precision of CRISPR–Cas systems as next-generation antimicrobial tools.

### 2.5 Data extraction and management

Data from included studies were extracted using a standardized form that captured the following parameters: CRISPR effector type and class (Cas9, Cas12, Cas13, etc.), bacterial target and resistance mechanism, delivery system (phage, conjugative plasmid, nanoparticle, etc.), antimicrobial mechanism, and main experimental outcomes.

### 2.6 Data synthesis

Due to heterogeneity in study designs, outcome measures, and delivery systems, a narrative synthesis approach was adopted instead of meta-analysis. Studies were grouped based on the main CRISPR mechanism (chromosomal targeting, plasmid elimination, biofilm inhibition) and the delivery platform (phage-based, conjugative plasmid, nanoparticle-based). The synthesis also incorporated comparisons between *in vitro* and *in vivo* findings and discussed translational challenges and safety considerations (Table 1).

**TABLE 1:** Classification of references by subject area.

Domain/sub-theme (*n* = studies)	Key themes	Representative references (first author, year)	Brief description/focus
Mechanisms of action (*n* = 33)	•Classification of CRISPR–Cas systems • Mechanisms of antimicrobial action • Design considerations for CRISPR antibacterials •Chromosomal targeting •Plasmid elimination •Biofilm and virulence targeting •Multiplex and combinatorial approaches	(Ahmed et al., 2024; Allemailem et al., 2024; Balcha and Neja, 2023; Bhatia et al., 2023; Bikard and Barrangou, 2017; Ganguly et al., 2024; Gupta et al., 2022; Hillary and Ceasar, 2023; Hsu et al., 2014; Javed et al., 2023; Kang et al., 2017; Kippnich et al., 2024; Koonin et al., 2023; Li et al., 2023; Liu and Doudna, 2020; Mayorga-Ramos et al., 2023; McCarty et al., 2020; O’Connell, 2019; Okesanya et al., 2025; Palacios Araya et al., 2021; Pickar-Oliver and Gersbach, 2019; Reshetnikov et al., 2022; Saeed et al., 2025; Skovmand et al., 2025; Tagliaferri et al., 2020; Van Beljouw et al., 2023; Wang et al., 2020; Wang et al., 2023; Wu et al., 2021; Yan et al., 2019; Yang and Patel, 2024; Zhang R. et al., 2024; Zhou et al., 2025)	•Classification of CRISPR–Cas systems: Two main classes (I & II) differing in effector complexity and DNA/RNA targeting. •Mechanisms of antimicrobial action: Uses Cas nucleases to cleave chromosomal, plasmid, or RNA targets for bacterial killing. •Design considerations: Emphasizes gRNA precision, nuclease selection, and multiplexing to enhance specificity and efficacy. •Chromosomal targeting: Induces lethal double-strand DNA breaks in essential bacterial genes. •Plasmid elimination: Removes resistance plasmids to resensitize bacteria to antibiotics. •Biofilm and virulence targeting: Disrupts quorum sensing and virulence gene expression to weaken infection. •Multiplex and combinatorial approaches: Combines multiple guides, Cas effectors, or antibiotics to prevent resistance escape.
Antimicrobial approaches using CRISPR-Cas (*n* = 8)	•Chromosomal targeting •Plasmid elimination •Biofilm and virulence disruption •Multiplex and combinatorial approaches	(Abudayyeh et al., 2016; Dong et al., 2019; Huan et al., 2023; Kiga et al., 2020; Mayorga-Ramos et al., 2023; Saffari Natanzi et al., 2025; Tao et al., 2023; Zhao et al., 2023)	•Chromosomal targeting: Lethal gene disruption in MDR bacteria through Cas9/Cas12 cleavage. •Plasmid elimination: Removal of resistance plasmids to restore antibiotic susceptibility. •Biofilm and virulence targeting: Interference with quorum sensing and virulence gene expression. •Multiplex approaches: Combined Cas systems or antibiotics to enhance efficacy and limit resistance.
Delivery and transmission strategies (*n* = 15)	•Bacteriophage/phagemid delivery •Conjugative plasmids •Nanoparticles/vesicles •Other delivery methods	(Onyeyili et al., 2025; Kim et al., 2024; Chaudhary et al., 2025; Ghaznavi et al., 2025; Nath et al., 2022; Pacia et al., 2024; Łobocka et al., 2021; Balcha and Neja, 2023; Huan et al., 2023; Tao et al., 2022; Jia et al., 2024; Saffari Natanzi et al., 2025; Thavorasak et al., 2025; Assad-Garcia et al., 2022; Nour El-Din et al., 2025)	•Bacteriophage/phagemid delivery: Combines phage specificity with CRISPR precision for targeted bacterial killing; advancing toward clinical use. •Conjugative plasmids: Enables horizontal transfer of CRISPR antimicrobials to eliminate resistant bacteria. •Nanoparticles/vesicles: Non-viral carriers improve CRISPR delivery efficiency in MDR pathogens. •Other methods: Experimental *in vitro* systems like electroporation or artificial phage-like particles.
*In vivo* preclinical studies and current status (*n* = 20)	•Animal models and gut microbiome •Other infection models •Pathogen-specific studies •Comparison with conventional therapies	(Ates et al., 2024; Baqar et al., 2024; Duggan and Mostowy, 2018; Gomes and Mostowy, 2020; Hamida et al., 2020; Haval et al., 2025; Huan et al., 2023; Junaid et al., 2023; Reymond et al., 2013; Rodrigues et al., 2019; Saffari Natanzi et al., 2025; Scoffone et al., 2025; Tyumentseva et al., 2021; Zhang et al., 2023; Wang J. et al., 2024; Pacia et al., 2024; Nath et al., 2022; Yeh et al., 2022; Kashyap et al., 2025)	•Animal models & gut microbiome: Demonstrates CRISPR delivery reducing resistant gut bacteria while maintaining microbiome stability. •Other infection models: Shows effective bacterial clearance and biofilm reduction in zebrafish and surface infection models. •Pathogen-specific studies: Targets virulence and resistance genes in key pathogens (*A. baumannii*, *S. aureus*) to restore antibiotic sensitivity. •Comparison with conventional therapies: Highlights CRISPR’s precision and specificity over phage and synthetic antimicrobials in combating resistance.
Barriers, risks, and resistance mechanisms (*n* = 24)	•Delivery challenges •Escape mutations and resistance •Off-target effects •Immunogenicity and safety •Regulatory and ethical concerns	(Akbarian and Chen, 2022; Anliker et al., 2022; Asmamaw Mengstie et al., 2024; Brenker et al., 2025; Du et al., 2023; Forsberg, 2023; Gleditzsch et al., 2019; Wang H. et al., 2024; Hille and Charpentier, 2016; Chen et al., 2017; Janik et al., 2020; Kadkhoda et al., 2024; Kazemian et al., 2022; Kolanu, 2024; Li and Bondy-Denomy, 2021; Mayorga-Ramos et al., 2023; Mir et al., 2022; Nath et al., 2022; Quinones-Olvera et al., 2024; Saffari Natanzi et al., 2025; Silva et al., 2016; Türeli and Türeli, 2020; West and Gronvall, 2020; Xuan et al., 2023)	•Delivery challenges: Limited host range, instability in complex environments, and manufacturing scale-up difficulties. •Escape mutations and resistance: Bacterial mutations, DNA repair, and anti-CRISPR proteins reduce CRISPR efficacy. •Off-target effects: Unintended edits or collateral cleavage affecting beneficial microbes and genetic stability. •Immunogenicity and safety: Potential immune reactions and toxicity from delivery systems in hosts. •Regulatory and ethical concerns: Biosafety, environmental containment, and governance challenges for CRISPR antimicrobials.
Future directions and research priorities (*n* = 27)	•Optimizing delivery platforms •Multiplexed targeting and anti-Acr strategies •Combination therapies •Amplified *in vivo* and translational research •Standardization and regulatory frameworks	(Ahmed et al., 2024; Allemailem et al., 2024; Durr and Leipzig, 2023; Escalona-Noguero et al., 2021; Ju and Sun, 2017; Kadkhoda et al., 2024; Chen et al., 2021; Laurent et al., 2024; Lenneman et al., 2021; McCarty et al., 2020; Marino et al., 2020; Najafi et al., 2022; Nazir et al., 2024; Ophinni et al., 2020; Pacesa et al., 2024; Palacios Araya et al., 2021; Park et al., 2025; Pawluk et al., 2016; Rabuma et al., 2024; Ramos et al., 2023; Rauch et al., 2017; Sharma et al., 2021; Shrestha et al., 2022; Strathdee et al., 2023; Tridgett et al., 2021; Vivekanandan et al., 2025; Yadav and Chauhan, 2024)	•Optimizing delivery platforms: Enhancing phage host range, genome capacity, and hybrid phage–nanoparticle delivery stability. •Multiplexed targeting and anti-Acr strategies: Using multiple gRNAs and Cas variants to prevent resistance and counter anti-CRISPR effects. •Combination therapies: Pairing CRISPR with antibiotics, phages, or immune modulators to boost antibacterial efficacy. •Amplified *in vivo* and translational research: Expanding studies into large animal and human microbiome models for safety and efficacy. •Standardization and regulatory frameworks: Developing protocols and biosafety regulations for CRISPR therapeutic evaluation and manufacturing.

This table summarizes all studies that met the inclusion criteria of this systematic review, categorized according to their primary research domain and thematic focus for narrative synthesis. A total of 127 references were identified, some references appeared in multiple thematic domains due to cross cutting relevance. Five introductory references were excluded from the tabulation. Each domain outlines key themes, representative studies, and their principal research focus, encompassing mechanisms of CRISPR–Cas antibacterial action, delivery strategies, preclinical and *in vivo* studies, barriers and risks, and future research priorities.

## 3 Results

The included studies were organized into six major thematic domains reflecting the scope and focus of CRISPR–Cas–based antibacterial research. Mechanisms of action represented the largest thematic domain. These studies described the classification of CRISPR–Cas systems into major classes and types, alongside their molecular mechanisms relevant to antibacterial activity. Reported mechanisms included chromosomal targeting through induction of double-strand DNA breaks in essential or resistance associated genes, elimination of resistance carrying plasmids, targeting of integron platforms, disruption of biofilm formation, and repression of virulence factor expression. Several studies also addressed design considerations such as guide RNA specificity, nuclease selection, and multiplex targeting strategies. Multiplex and combinatorial approaches involving multiple guide RNAs, distinct Cas effectors, or adjunct antimicrobial agents were frequently reported.

A subset of studies specifically focused on antimicrobial approaches using CRISPR–Cas systems. These investigations demonstrated chromosomal targeting for bactericidal activity, plasmid elimination to restore antibiotic susceptibility, biofilm and virulence disruption, and the use of multiplex strategies to enhance antibacterial efficacy and reduce resistance emergence.

Delivery and transmission strategies constituted another major domain. Reported delivery platforms included bacteriophage or phagemid based systems, conjugative plasmids, nanoparticle- and vesicle-based carriers, and alternative experimental delivery methods. Phage and phagemid systems were commonly used to exploit host specificity, while conjugative plasmids enabled horizontal dissemination of CRISPR constructs among bacterial populations. Non-viral systems such as nanoparticles and vesicles were explored as carriers to improve delivery efficiency and stability, whereas other methods were primarily applied in controlled *in vitro* settings.

*In vivo* preclinical studies and current status employed animal models and gut microbiome systems, alternative infection models, and pathogen specific approaches. Reported outcomes included reduction of resistant bacterial populations in the gut with limited microbiome disruption, effective bacterial clearance and biofilm reduction in infection models, and restoration of antibiotic susceptibility in clinically relevant pathogens. Comparative assessments with conventional antimicrobial strategies were also reported within this domain.

Barriers, risks, and resistance mechanisms which identified challenges included limitations in delivery efficiency and host range, instability of CRISPR systems in complex biological environments, and difficulties related to manufacturing and scalability. Resistance mechanisms such as target mutations, DNA repair responses, and anti-CRISPR proteins were documented. Additional concerns included off target activity, collateral nucleic acid damage, immunogenicity of delivery systems, and biosafety, regulatory, and ethical considerations.

Future directions and research priorities focused on optimizing delivery platforms, developing multiplexed targeting strategies and anti-anti-CRISPR approaches, combining CRISPR antimicrobials with antibiotics or other therapies, expanding *in vivo* and translational research, and establishing standardized evaluation protocols and regulatory frameworks.

## 4 Discussion

### 4.1 Mechanisms and types of CRISPR-Cas systems of importance to antibacterial strategies

#### 4.1.1 Classification of CRISPR-Cas systems

CRISPR–Cas systems are broadly divided into two major classes, Class I and Class II, which differ fundamentally in effector composition and molecular execution. Class I systems (e.g., Type I and Type III) rely on multi-protein complexes such as Cascade with Cas3 mediating processive DNA degradation, whereas Class II systems employ single, multifunctional effector proteins such as Cas9 (Type II), Cas12 (Type V), and Cas13 (Type VI) to independently recognize and cleave nucleic acid targets (Liu and Doudna, 2020; Ganguly et al., 2024).

Beyond the extensively characterized Cas9, alternative effectors substantially broaden the CRISPR toolbox. DNA targeting Cas12 variants (Cas12a/Cas12b/Cas12f) recognize T-rich PAMs and generate staggered double strand breaks with collateral single stranded DNA cleavage, supporting genome editing, transcriptional regulation, and sensitive diagnostic platforms such as DETECTR across diverse biological systems (Hillary and Ceasar, 2023; Zhang R. et al., 2024; Yan et al., 2019; Koonin et al., 2023). Cas13 (Type VI), by contrast, targets RNA and mediates collateral RNase activity, enabling programmable RNA knockdown, editing, imaging, and SHERLOCK based diagnostics in infectious disease and oncology (Yang and Patel, 2024; Wang et al., 2023; Wei et al., 2021; Pickar-Oliver and Gersbach, 2019; Wang et al., 2020; Zhou et al., 2025; Gupta et al., 2022). Ultra compact nucleases such as Cas14 and related miniature Cas12 variants further extend this diversity, offering advantages for point of care diagnostics and *in vivo* delivery due to their small size and ssDNA targeting capacity (Hillary and Ceasar, 2023; Yan et al., 2019; Li et al., 2023; Wu et al., 2024).

From an antimicrobial perspective, this functional diversity underscores that CRISPR–Cas systems are not interchangeable tools but context-dependent platforms whose molecular architecture directly shapes therapeutic feasibility, delivery constraints, and safety profiles. Collectively, these emerging effectors expand CRISPR applications well beyond Cas9, spanning diagnostics and programmable RNA therapeutics, while simultaneously introducing translational challenges related to cargo size, PAM constraints, off-target and collateral cleavage, immunogenicity, biosafety, and long-term stability that must be addressed before clinical deployment (Hillary and Ceasar, 2023; Wang et al., 2023; Pickar-Oliver and Gersbach, 2019; Koonin et al., 2023; Zhou et al., 2025; Bhatia et al., 2023).

Accordingly, nuclease selection in antimicrobial development should be viewed as a strategic decision balancing antibacterial potency with translational practicality, particularly in complex microbial communities rather than isolated pathogens.

#### 4.1.2 Mechanisms of action in antimicrobial usage

CRISPR–Cas antimicrobials resensitize multidrug-resistant (MDR) bacteria through a spectrum of genetic interventions targeting chromosomal loci, plasmids, integrons, and RNA, thereby extending beyond conventional bactericidal paradigms. Rather than acting solely as killing agents, these systems function as precision tools that reprogram bacterial susceptibility landscapes.

Chromosomal targeting using Cas9 (and in some studies Cas12) induces lethal double-strand breaks (DSBs) in resistance or essential genes, exploiting the limited DNA repair capacity of most bacteria and enabling selective killing or resensitization when genes such as *mecA, mcr-1, bla_*KPC*_, tetM*, and *ermB* are disrupted (Mayorga-Ramos et al., 2023; Allemailem et al., 2024; Palacios Araya et al., 2021; Zhang H. et al., 2024; Javed et al., 2023; Ahmed et al., 2024; Saeed et al., 2025). This approach highlights the unique advantage of CRISPR systems in converting resistance determinants into intrinsic liabilities rather than relying on broad-spectrum toxicity.

Plasmid focused strategies similarly restore antibiotic susceptibility by curing resistance bearing plasmids using Cas9 or type I systems, even in high copy contexts when multiple targets or essential plasmid functions (e.g., *repA/repB/parA*, class 1 *integron intI1*) are disrupted (Mayorga-Ramos et al., 2023; Kippnich et al., 2024; Van Beljouw et al., 2023; Wu et al., 2021; Tagliaferri et al., 2020; Ahmed et al., 2024; Skovmand et al., 2025). Importantly, integron targeting represents a systems-level intervention capable of collapsing entire multidrug resistance cassettes in a single event, while dCas9-based CRISPRi enables reversible transcriptional repression of chromosomal or plasmid-borne AMR genes without inducing DNA cleavage (Mayorga-Ramos et al., 2023; Allemailem et al., 2024; Palacios Araya et al., 2021; Wu et al., 2021; Skovmand et al., 2025).

RNA targeting systems such as Cas13 introduce a mechanistically distinct modality, whereby phage-delivered Cas13a constructs (CapsidCas13a) eliminate bacteria by recognizing AMR transcripts regardless of genomic location, coupling sequence-specific RNA targeting with collateral RNA degradation (Mayorga-Ramos et al., 2023; Kang et al., 2017; Van Beljouw et al., 2023; O’Connell, 2019; Yang and Patel, 2024). This RNA-centric strategy is particularly well suited to rapidly evolving resistance landscapes, as it bypasses permanent genomic modification and enables adaptive targeting of expressed resistance phenotypes.

Across these approaches, reported resensitization efficiencies range from ∼5–100%, reflecting strong dependence on target selection, delivery modality (conjugative plasmids, phages/phagemids, nanoparticles, electroporation), and plasmid copy number rather than nuclease activity alone (Allemailem et al., 2024; Kippnich et al., 2024; Kang et al., 2017; Van Beljouw et al., 2023; Zhang H. et al., 2024; Wu et al., 2021; Okesanya et al., 2025; Ahmed et al., 2024). This variability underscores that ecological context and delivery efficiency are dominant determinants of therapeutic success.

Consequently, key translational challenges remain, including effective delivery into diverse and intracellular pathogens, escape via spacer or target mutation and anti-CRISPR activity, management of collateral nucleic acid damage (particularly with Cas13 and Cas12), and preservation of beneficial microbiota while achieving durable resistance suppression (Mayorga-Ramos et al., 2023; Allemailem et al., 2024; Kang et al., 2017; Balcha and Neja, 2023; Van Beljouw et al., 2023; Bikard and Barrangou, 2017; Ahmed et al., 2024). Addressing these barriers will ultimately determine whether CRISPR antimicrobials can transition from experimental tools to clinically reliable resistance modifying therapies.

#### 4.1.3 Design considerations for CRISPR-based antibacterials

Effective deployment of CRISPR-based antimicrobial systems depends on careful optimization of multiple interdependent design parameters. Guide RNA (gRNA) design is central to achieving high target specificity and minimizing off-target cleavage, with preference for conserved regions within resistance or virulence genes and, where appropriate, multiplexed gRNAs to enhance robustness. The choice of nuclease is similarly context-dependent, influenced by whether DNA or RNA targeting is required, PAM constraints, enzyme size, immunogenic potential, and compatibility with the intended delivery platform (Hsu et al., 2014; Reshetnikov et al., 2022).

From a translational perspective, these variables collectively determine not only molecular efficacy but also therapeutic selectivity, durability of resistance suppression, and safety in complex microbial ecosystems. Multiplexed targeting, simultaneous engagement of multiple genes or loci, further strengthens antibacterial activity by reducing the likelihood of resistance escape and buffering against target variability (McCarty et al., 2020). Together, these considerations emphasize that clinically viable CRISPR antimicrobials will require integrated, systems-level design strategies rather than optimization of individual components in isolation.

### 4.2 Antimicrobial approaches using CRISPR–Cas

Antimicrobial strategies based on CRISPR–Cas systems exploit programmable nucleases to selectively eliminate multidrug-resistant bacteria or disable resistance and virulence determinants through chromosomal targeting, plasmid curing, RNA interference, and combinatorial approaches. Taken together, these modalities illustrate a paradigm shift from broad-spectrum bacterial suppression toward precision genetic interventions tailored to specific resistance architectures.

#### 4.2.1 Chromosomal targeting and bactericidal killing

Targeting essential chromosomal genes enables direct bactericidal activity, as demonstrated by phage-based delivery of Cas9 against *Shigella flexneri*, which reduced bacterial burden and improved host survival *in vivo* (Huan et al., 2023). Such approaches highlight the potential of CRISPR to function as a sequence-specific antimicrobial, although their clinical applicability will depend on balancing lethality with delivery efficiency and microbiome preservation.

#### 4.2.2 Plasmid elimination and resensitization

Elimination of resistance plasmids encoding β-lactamases, *mcr* genes, ESBLs, or carbapenemases represents a central CRISPR strategy, with conjugative systems successfully reducing resistant *Enterococcus faecalis* populations *in vitro* and in murine gut models (Dong et al., 2019; Tao et al., 2023). Importantly, plasmid curing reframes antimicrobial therapy as resistance reversal rather than bacterial eradication, potentially extending the lifespan of existing antibiotics.

#### 4.2.3 Targeting biofilms and virulence factors

Because biofilms confer protection from antibiotics and immune responses, CRISPR mediated disruption of quorum sensing, matrix production, or regulatory pathways offers a means to sensitize bacteria within structured communities. RNA targeting systems such as Cas13 further enable suppression of virulence-associated transcripts (Abudayyeh et al., 2016; Kiga et al., 2020; Saffari Natanzi et al., 2025). These strategies underscore CRISPR’s capacity to attenuate pathogenicity without necessarily imposing strong selective pressure for survival based resistance.

#### 4.2.4 Multiplex and combinatorial approaches

Combining CRISPR systems with antibiotics, dual gRNAs, or multiple Cas effectors enhances antibacterial efficacy and reduces escape potential, particularly in biofilms or mixed microbial populations (Mayorga-Ramos et al., 2023; Zhao et al., 2023). Collectively, these findings suggest that CRISPR antimicrobials are most effective when deployed as part of integrated, multi-modal treatment strategies rather than as standalone interventions.

### 4.3 Delivery platforms: current methods, advantages, and limitations

#### 4.3.1 Bacteriophage/phagemid delivery

Bacteriophages represent a promising delivery platform for CRISPR based antimicrobials, leveraging their natural ability to introduce genetic material into bacteria. Preclinical studies demonstrate that phages or phagemids carrying CRISPR–Cas payloads can effectively eliminate bacterial pathogens *in vivo* (Onyeyili et al., 2025). From a therapeutic standpoint, the key strength of phage-mediated delivery lies in the convergence of biological host specificity with programmable genetic targeting, enabling selective eradication of resistant strains while largely preserving commensal microbiota.

Phages also offer practical advantages in complex environments such as biofilms and mucosal surfaces, where conventional antibiotics often fail. However, these advantages are counterbalanced by translational constraints that may ultimately define clinical feasibility, including restricted host range, immune-mediated clearance, lysogenic conversion risks, challenges in stable encapsidation of CRISPR payloads, and the presence of anti-CRISPR proteins that can neutralize nuclease activity. Collectively, these limitations suggest that phage delivery is most suitable for precision, pathogen-specific interventions rather than broadly deployable antimicrobial platforms.

Clinical progress with engineered bacteriophages has strengthened the translational relevance of this approach. The Phase 2 ELIMINATE trial evaluated LBP-EC01, a genetically enhanced six-phage cocktail for treatment of *Escherichia coli* urinary tract infection, independent of resistance status (Kim et al., 2024). Combined intraurethral and intravenous administration with oral trimethoprim sulfamethoxazole was well tolerated and achieved early and sustained bacterial clearance with complete symptom resolution (NCT05488340). Although this trial did not directly evaluate CRISPR encoded phages, it provides critical clinical proof-of-concept for the safety, manufacturability, and regulatory acceptance of genetically engineered phage therapeutics, thereby lowering translational barriers for future CRISPR-enabled phage platforms (Kim et al., 2024).

#### 4.3.2 Current human applications and translational progress

Although most CRISPR–Cas based antibacterial systems remain in preclinical development, progress in engineered bacteriophage therapies has brought related technologies closer to clinical translation (Chaudhary et al., 2025; Ghaznavi et al., 2025). This disparity highlights that delivery feasibility, rather than nuclease functionality, currently represents the primary bottleneck for clinical advancement of CRISPR antimicrobials. While no CRISPR–Cas antibacterial therapy has yet entered human trials, phage-based interventions enhanced through genetic manipulation are already being evaluated clinically (Nath et al., 2022; Pacia et al., 2024).

The LBP-EC01 bacteriophage cocktail developed by Locus Biosciences demonstrated safety, tolerability, and rapid *E. coli* eradication in uncomplicated urinary tract infections (ELIMINATE trial, NCT05488340), with comparable efforts such as Armata Pharmaceuticals’ AP-PA02 targeting *Pseudomonas aeruginosa* infections. These studies collectively signal a broader translational shift toward precision bacteriophage therapeutics, establishing regulatory and clinical precedents that are directly relevant to future CRISPR encoded antimicrobial platforms. Rather than end points themselves, these trials function as enabling milestones for CRISPR based antibacterial strategies in human medicine.

Preclinical and early clinical data further support the viability of bacteriophage mediated delivery systems as a bridge toward CRISPR antimicrobial deployment. For example, P1 phage mediated *in vivo* delivery of Cas9 phagemids into *Shigella flexneri* significantly reduced bacterial load in zebrafish larvae models (Łobocka et al., 2021; Balcha and Neja, 2023; Huan et al., 2023). Together, these findings suggest that the true translational value of phage-CRISPR systems lies not only in antimicrobial efficacy, but in their potential to redefine antibacterial therapy as a programmable, pathogen-specific intervention rather than a broadly cytotoxic approach to infection control.

#### 4.3.3 Conjugative plasmids/mobilizable genetic elements

This approach exploits plasmids that naturally transfer between bacteria to disseminate CRISPR antimicrobials, as demonstrated in *Enterococcus* models where delivery to the gut microbiota reduced antibiotic-resistant populations (Tao et al., 2022). From a translational perspective, conjugative systems are particularly compelling because they harness endogenous bacterial communication networks, enabling population-level spread of CRISPR activity without reliance on external vectors. However, this same property raises important considerations regarding ecological control, specificity, and containment, especially within complex microbial communities.

#### 4.3.4 Nanoparticles, vesicles, hybrid systems

Non-viral delivery platforms, including biomimetic vesicles, cationic vesicles, and liposomes, have been explored for delivering CRISPR components as DNA, RNA, or ribonucleoprotein complexes (Jia et al., 2024). Biomimetic cationic hybrid vesicles carrying CRISPR/Cas9 plasmids have shown *in vivo* efficacy against *Acinetobacter baumannii* and *P. aeruginosa* (Jia et al., 2024; Saffari Natanzi et al., 2025; Thavorasak et al., 2025). These systems conceptually decouple CRISPR delivery from biological replication, offering greater modularity and biosafety control compared with phage-based approaches, albeit at the cost of reduced targeting specificity and penetration efficiency in some infection contexts.

#### 4.3.5 Other methods

Electroporation and chemical modification remain efficient for *in vitro* delivery but are not readily translatable *in vivo*. In parallel, cell free systems and artificial phage-like particles are being explored as alternative delivery strategies (Assad-Garcia et al., 2022; Nour El-Din et al., 2025). While currently experimental, these platforms illustrate an emerging design philosophy that prioritizes controllability, manufacturability, and regulatory compatibility over maximal delivery efficiency, which may ultimately shape clinically acceptable CRISPR antimicrobial formats.

### 4.4 *In vivo* preclinical studies and current status

#### 4.4.1 Animal models and gut microbiome

Preclinical studies consistently demonstrate that CRISPR delivery systems can reduce antibiotic-resistant bacteria within the gut. For example, conjugative plasmid-mediated CRISPR delivery in mouse intestines markedly reduced resistant *Enterococcus faecalis* populations (Rodrigues et al., 2019), while phage-delivered CRISPR approaches minimized multidrug-resistant pathogens with negligible disruption of microbiome stability (Nath et al., 2022; Zhang et al., 2023). Collectively, these findings suggest that CRISPR antimicrobials can achieve resistance suppression while preserving commensal community structure, a critical advantage over broad-spectrum antibiotics in microbiome sensitive environments.

#### 4.4.2 Other infection models

Biofilm and animal infection models further support the therapeutic potential of CRISPR based antimicrobials. Phage-mediated Cas9 delivery targeting *Shigella flexneri* in zebrafish larvae resulted in strong bactericidal activity and improved host survival (Duggan and Mostowy, 2018; Gomes and Mostowy, 2020; Huan et al., 2023). Similarly, in biofilm and surface infection models, the spatio-temporal co-delivery of CRISPR systems with vesicles or nanoparticles reduced biofilm biomass and increased bacterial susceptibility in *Klebsiella* and *Pseudomonas* species (Hamida et al., 2020; Reymond et al., 2013; Saffari Natanzi et al., 2025; Haval et al., 2025). These models highlight that CRISPR efficacy is strongly context-dependent, with spatial delivery and local microenvironment emerging as key determinants of antibacterial outcome.

#### 4.4.3 Pathogen-specific studies

Recent work increasingly applies CRISPR based strategies to high-priority pathogens. In *Acinetobacter baumannii*, gene-editing approaches have been used to attenuate virulence and resistance traits (Tyumentseva et al., 2021; Junaid et al., 2023; Baqar et al., 2024; Scoffone et al., 2025). Similarly, in *Staphylococcus aureus*, CRISPR-Cas9 targeting of resistance genes such as *mecA* has restored methicillin susceptibility (Ates et al., 2024; Wang J. et al., 2024). Together, these pathogen-focused studies suggest that CRISPR antimicrobials may be most impactful when tailored to organism-specific resistance architectures rather than deployed as universal agents.

To provide a structured overview of the available *in vivo* and preclinical evidence, Table 2 summarizes representative studies on CRISPR-based antimicrobials, including model systems, delivery strategies, bacterial targets, and main outcomes.

**TABLE 2:** Summary of representative *in vivo* and preclinical CRISPR–Cas antimicrobial studies.

Model/system	Pathogen(s)	Delivery strategy	CRISPR target/mechanism	Main outcome	References
Mouse gut model	*Enterococcus faecalis*	Conjugative plasmid	CRISPR-Cas9 plasmid curing	Reduced resistant populations, restored sensitivity	(Rodrigues et al., 2019)
Mouse gut model	MDR gut bacteria	Phage delivery	Cas9-mediated AMR targeting	↓ MDR load, minimal microbiome disruption	(Nath et al., 2022; Zhang et al., 2023)
Zebrafish larvae	*Shigella flexneri*	Phage (P1-based)	Cas9 chromosomal targeting	Reduced bacterial load, improved survival	(Duggan and Mostowy, 2018; Huan et al., 2023; Gomes and Mostowy, 2020)
Murine biofilm model	*Klebsiella, Pseudomonas*	Nanoparticles/vesicles	Cas9, Cas13 targeting of biofilm genes	↓ Biofilm biomass, enhanced susceptibility	(Hamida et al., 2020; Saffari Natanzi et al., 2025; Reymond et al., 2013; Haval et al., 2025)
Murine infection model	*Acinetobacter baumannii*	Plasmid or phage	Gene editing of virulence/AMR genes	↓ Resistance, ↓ virulence	(Tyumentseva et al., 2021; Junaid et al., 2023; Baqar et al., 2024; Scoffone et al., 2025)
Murine infection model	*Staphylococcus aureus*	Cas9 system	Targeting mecA resistance gene	Restored methicillin sensitivity	(Ates et al., 2024; Wang J. et al., 2024)

#### 4.4.4 Comparison of CRISPR-based antimicrobials with conventional and emerging therapies

Compared with conventional antibacterial strategies, CRISPR based antimicrobials offer unparalleled precision by directly targeting defined resistance determinants. Both CRISPR systems and phage therapy are more selective than synthetic antimicrobials, which broadly suppress microbial populations and risk collateral damage and resistance emergence (Pacia et al., 2024; Yeh et al., 2022). CRISPR enables direct genomic modification of bacteria, whereas phage therapy relies on viral infection and lysis (Nath et al., 2022). This distinction positions CRISPR as a fundamentally different therapeutic paradigm, one centered on genetic reprograming rather than microbial eradication, while phage therapy leverages biological predation. Although both approaches hold promise, CRISPR’s genome-editing precision may confer greater long-term control over resistance, whereas phage therapy benefits from natural amplification and host specificity (Kashyap et al., 2025).

### 4.5 Barriers, risks, and resistance mechanisms

#### 4.5.1 Delivery challenges

CRISPR delivery systems face practical constraints, including limited host range, as phages or conjugative plasmids may not efficiently target all clinically relevant bacterial strains (Quinones-Olvera et al., 2024). Their stability can also be compromised in complex biological environments such as biofilms, during host immune responses, across variable physiological pH, or following nuclease exposure (Silva et al., 2016; Akbarian and Chen, 2022). In addition, translation to clinical or commercial settings requires careful upscaling under Good Manufacturing Practice (GMP) conditions to ensure reproducible manufacturing, packaging, and safe delivery (Türeli and Türeli, 2020). Together, these challenges indicate that delivery, not nuclease efficiency, remains the primary bottleneck for clinical translation, underscoring the need for platform-agnostic delivery optimization rather than incremental improvements in CRISPR activity alone.

#### 4.5.2 Escape mutations and resistance

Bacteria can evade CRISPR activity through multiple mechanisms, including point mutations in protospacer or PAM sequences, deletion or loss of target genes or plasmids, and activation of DNA repair pathways such as homologous recombination that counteract lethal double-strand breaks (Hille and Charpentier, 2016; Gleditzsch et al., 2019; Mayorga-Ramos et al., 2023; Du et al., 2023). In addition, many bacteriophages and mobile genetic elements encode anti-CRISPR (Acr) proteins that directly inhibit CRISPR associated nucleases such as Cas9 and Cas12 (Li and Bondy-Denomy, 2021; Forsberg, 2023; Kadkhoda et al., 2024; Brenker et al., 2025). These escape pathways mirror evolutionary dynamics observed with antibiotics and phages, suggesting that CRISPR antimicrobials will require multiplexed targeting and adaptive designs to achieve durable efficacy in real-world microbial ecosystems.

#### 4.5.3 Non-specific, collateral damage to cellular mechanisms

CRISPR technologies carry risks of off-target effects that may inadvertently damage beneficial commensal bacteria and disrupt microbiome homeostasis (Kolanu, 2024; Chen et al., 2017). Moreover, collateral cleavage activity of certain CRISPR effectors can lead to non-specific genomic degradation, while unintended release or horizontal transfer of plasmids or CRISPR constructs may facilitate gene spread among microbial communities (Asmamaw Mengstie et al., 2024; Saffari Natanzi et al., 2025). These risks highlight that antimicrobial precision at the genetic level does not automatically translate to ecological precision, emphasizing the importance of microbiome aware therapeutic design.

#### 4.5.4 Immunogenicity and safety in hosts

Phage or phage mediated CRISPR delivery may trigger immune clearance or inflammatory responses in hosts (Nath et al., 2022; Wang H. et al., 2024). Additionally, nanoparticle, and vesicle, based delivery systems present variable toxicity profiles, and uncertainties remain regarding biodistribution, pharmacokinetics, and long-term persistence of CRISPR reagents *in vivo* (Kazemian et al., 2022; Xuan et al., 2023). These safety considerations underscore the need for comprehensive immunological and toxicological profiling before CRISPR antimicrobials can advance into human trials.

#### 4.5.5 Regulatory, ethical, and ecological concerns

Regulatory frameworks for CRISPR based antimicrobials remain underdeveloped, as these technologies intersect characteristics of biological agents, live phage therapies, and gene-editing tools, creating complex governance challenges (Anliker et al., 2022). Their use also raises ethical concerns related to biosafety, environmental containment, unintended gene dissemination, and dual-use potential (West and Gronvall, 2020; Janik et al., 2020; Mir et al., 2022).

Addressing these issues early through coordinated regulatory, ethical, and ecological oversight will be essential to prevent translational delays and public resistance as CRISPR antimicrobials move toward clinical application.

### 4.6 Future directions and research priorities

#### 4.6.1 Optimizing delivery platforms

Phage engineering efforts increasingly focus on expanding host range, eliminating undesirable genetic elements, and improving genome payload size and packaging efficiency (Lenneman et al., 2021; Durr and Leipzig, 2023). In parallel, synthetic or phage-mimicry particles and engineered vesicles with enhanced stability in host environments are being developed, including hybrid systems that integrate phage specificity with nanoparticle versatility (Ju and Sun, 2017; Chen et al., 2021; Tridgett et al., 2021). These advances collectively suggest that future success will depend less on identifying a single optimal carrier and more on tailoring delivery platforms to infection site, pathogen ecology, and treatment duration.

#### 4.6.2 Multiplexed targeting and anti-Acr strategies

To mitigate viral escape driven by single-point mutations, multiplex guide RNAs and multi Cas effector strategies are increasingly employed (McCarty et al., 2020; Ophinni et al., 2020; Escalona-Noguero et al., 2021; Najafi et al., 2022). In addition, naturally less Acr-sensitive Cas proteins or engineered variants resistant to Acr suppression are being explored (Pawluk et al., 2016; Rauch et al., 2017; Marino et al., 2020; Allemailem et al., 2024; Kadkhoda et al., 2024).

These approaches reflect a shift toward evolutionary-informed CRISPR design, anticipating bacterial and phage countermeasures rather than responding to resistance *post-hoc*.

#### 4.6.3 Combination therapies

CRISPR antimicrobials are being investigated in combination with conventional antibiotics to resensitize resistant bacteria (Palacios Araya et al., 2021; Vivekanandan et al., 2025), as well as with phage therapy, immune modulators, and biofilm-disrupting agents to enhance antibacterial efficacy while limiting resistance emergence (Shrestha et al., 2022; Strathdee et al., 2023; Park et al., 2025).

Such combinatorial strategies position CRISPR systems as modulators of therapeutic response rather than standalone replacements for existing antimicrobials.

#### 4.6.4 Amplified *in vivo* and translational research

Future studies must extend beyond proof-of-concept experiments toward large-animal models and rigorously designed clinical trials (Sharma et al., 2021; Laurent et al., 2024; Pacesa et al., 2024). Long term investigations within relevant human microbiomes, including gut, skin, and lung, are also essential to assess safety, stability, and therapeutic durability (Nazir et al., 2024; Yadav and Chauhan, 2024) This progression is critical for defining realistic therapeutic windows and understanding long-term ecological consequences of CRISPR based interventions.

#### 4.6.5 Standardization, regulatory, and manufacturing frameworks

Establishing standardized protocols for evaluating efficacy, off target activity, and resistance escape is a pressing priority for CRISPR-based antimicrobials (Ahmed et al., 2024). These efforts must be aligned with clear regulatory frameworks addressing biosafety, environmental release, and intellectual property considerations associated with development and deployment (Ramos et al., 2023; Rabuma et al., 2024. Without early harmonization of regulatory and manufacturing standards, translational advances risk fragmentation and delayed clinical adoption despite technological readiness.

## 5 Conclusion

CRISPR-Cas systems hold a new frontier for the fight against multidrug-resistant bacteria. As pathogen-killing tools with precision, as Editors of resistance genes, and as reducers of collateral damage to commensal microbes, they could offer a potent adjunct or alternative to conventional antibiotics. However, significant technical and regulatory hurdles persist: delivery remains the major challenge; escape by bacteria and anti-CRISPR activity undermines durability; safety, immunogenicity, and environmental impact must be fully investigated. Success will likely depend on combined approaches: combining CRISPR with other antimicrobials, multiplex targeting, and rigorous translational models. With hard work, CRISPR antimicrobials can enter the therapeutic arsenal in clinical practice.

## Data Availability

The original contributions presented in this study are included in this article/supplementary material, further inquiries can be directed to the corresponding author.
